# Beyond entertainment: how intangible cultural heritage in video games shapes youth cultural identity

**DOI:** 10.3389/fpsyg.2026.1855866

**Published:** 2026-06-26

**Authors:** Yufang Chen, Zhaojun Wang, Jiali Chen, Chao Guo, Yang Wang, Yanxiang Hou

**Affiliations:** 1 School of Business, Shandong Xiehe University, Jinan, China; 2 School of Management Engineering, Shandong Jianzhu University, Jinan, China; 3 Business Innovation Department, University of the West of Scotland, Paisley, United Kingdom

**Keywords:** cultural identity, intangible cultural heritage (ICH), national culture, video games, young adults

## Abstract

While video games have traditionally been regarded as entertainment media, their role has expanded to encompass cultural dissemination and transmission. This study investigated how the integration of intangible cultural heritage elements within video games positively influences the development of cultural identity among the young generation. Data were collected via an online questionnaire completed by 513 young participants in China. Regression analysis and cluster analysis were employed to examine the underlying mechanisms. There are several main findings: (1) The degree of intangible cultural heritage embedded in video games exhibits a significant positive correlation with the enhancement of cultural identity attitudes and behaviors among young populations, where deeper integration yields stronger effects; (2) cultural identity attitude plays the mediating role between intangible cultural heritage embeddedness and cultural identity behavior; and (3) the impact of intangible cultural heritage embeddedness varies across different youth groups, as younger players demonstrate a higher level of responsiveness than the older players. These findings highlight video games' significant capacity to foster cultural identity among youth.

## 1 Introduction

According to a second-quarter 2025 global survey by Statista on internet user behavior, over 92% of respondents worldwide reported playing video games, and more than 83% of global internet users can be classified as gamers (Statista, 2025). In recent years, with the rapid advancement of digital technology, the gaming industry has experienced vigorous growth, quickly becoming an important part of the cultural and creative industries. Video games, leveraging the unique characteristics of the internet medium, have become a new carrier and form of expression for cultural dissemination and preservation, value transmission, and national image building (Jiang Q. et al., 2025; Yuan, 2024).

Many countries are increasingly incorporating video games into their cultural strategies, exploring how to achieve cultural exports and shape national images through game content design, market promotion, and narrative strategies. The United States targets youth, using game design to shape the thinking patterns of young players, thereby fostering an identification with American culture. At the same time, it leverages online markets and technological means to enhance immersion, allowing players to experience American values within a virtual world. Additionally, military-themed games subtly convey ideologies by letting players assume the role of American soldiers, reinforcing national narratives. Japan, on the other hand, presents its own history and modern life in a realistic manner, localizing European cultural elements to form a unique cultural expression, thus enhancing its cultural appeal. China, by integrating real-world natural landscapes, traditional festivals, and various intangible cultural heritages into game environments, allows players to experience the unique charm of Chinese culture within a virtual world.

The realization of cross-cultural transmission in games is closely related to their diverse multicultural content, the use of non-verbal symbols, player interaction, and extrinsic communication strategies. Specifically, culture is embedded in games through the translation of cultural elements (Rizvic et al., 2019), drawing inspiration from literary and artistic works (King et al., 2016), and integrating cultural connotations (Champion, 2020). Simultaneously, cultural elements within games are presented through visual representations, shaping typical cultural styles in interfaces via character design, interactive experiences, and scene creation (Muriel and Crawford, 2018). Additionally, leveraging the power of social media and player communities, cultural expressions in games transition from one-way dissemination to two-way interaction, fostering widespread cultural resonance (Ying and Li, 2025).

As the representative of the excellent traditional cultures, intangible cultural heritage (ICH) are vivid witnesses to the continuous inheritance of human civilization, serving as crucial links to bind national sentiments and construct cultural identity (Qiu et al., 2024). Governments, enterprises, and cultural institutions in various countries are actively exploring integration paths between “games” and “ICH” (Zeiler and Mukherjee, 2021), using visualization to reconstruct memories (Nusran and Zin, 2010) and promote the dynamic inheritance (Rattanarungrot et al., 2024) and international dissemination (Handrahan, 2020) of ICH. For instance, China has integrated paper-cutting art, Peking Opera art, lion dance art, and Nuo drama into Genshin Impact (Cheng, 2022).

Some scholars have conducted the theoretical discussion on the psychological and physiological engagement of young players with video game media, including their role in shaping cultural identity experiences (Chen and Qin, 2024). Jiang Y. et al. (2025) examines the cultural experiences embedded within games and their potential to foster cultural identity. However, empirical research specifically examining whether and how ICH in video games influences the cultural identity of young players remains limited and requires further in-depth exploration. This gap highlights the need for more systematic empirical study.

Therefore, this study seeks to answer whether there is a significant relationship between the ICH embeddedness in video games and ethnic cultural identity among young people? And do these effects vary across different youth groups. The following hypotheses are formulated to test this question. H1: ICH embeddness in video games is positively associated with player's cultural identity behavior. H2: Cultural identity attitude mediates therelationship between ICH embeddness and cultural identity behavior. H3: Player's characteristics moderates the positive effect of ICH embeddness on cultural identity behavior.

## 2 Materials and methods

### 2.1 Research design and data collection

This study collects data through web-based questionnaires conducted in Shandong Province, China. This study provided all participants with a clear explanation of the research purpose, content, and data usage before conducting the questionnaire. Participants gave their electronic informed consent and confirmed their voluntary participation. They were also informed that they could withdraw at any time. All data were anonymized and used solely for this research, in accordance with relevant ethical review requirements.The questionnaire comprised two main sections: demographic and gaming engagement questions, instruments measuring the core theoretical variables. A stratified sampling approach was employed to ensure representativeness. Specifically, young residents of Shandong aged between 18 and 34 were targeted, reflecting the primary demographic of interest in this research. The initial recruitment yielded 604 participants through multi-stage probability sampling. After excluding 91 cases that were ineligible due to age criteria or failed attention checks, our analysis cohort ultimately included 513 participants. These participants ranged in age from 18 to 34 years (M = 23.4, SD = 1.73), with a nearly equal gender distribution (279 females; 54.38%).

The empirical analysis design consists of three parts. Firstly, we examine the effect of the ICH embeddedness on cultural identity, including both cultural identity attitude and behavior. Secondly, we cluster young people into distinct groups based on their demographic characteristics and gaming engagement patterns. Thirdly, we analyze the heterogeneity of these effects across different youth groups.

### 2.2 Demographic and gaming engagement questions

Sociodemographic variables included gender, age, educational level, and occupation. Game engagement questions consisted of a range of behavioral indicators, such as spending on video games, gaming frequency and average weekly duration, the number of frequently played video games, total exposure time to video gaming, and maximum continuous gaming duration. The design of these measurement drew upon prior research on video games (Hagen et al., 2020; Wickham et al., 2020) with contextual modifications implemented to align with the specific cultural and methodological requirements of this investigation. Contextual adaptations were made to ensure cultural relevance and methodological suitability for the target population in this study.

### 2.3 Instruments

#### 2.3.1 Intangible cultural heritage embeddness

Cultural embedding primarily refers to the role of shared values in shaping economic strategies and objectives. In simpler terms, “cultural embedding” involves infusing new culture into a specific carrier or context, thereby shaping its internal characteristics.

Drawing on both established theoretical frameworks and empirical instruments, the scale measuring the *ICH Embeddness* comprises four items. On the one hand, the conceptual foundation is informed by previous theories of cultural embeddedness (Zukin and Dimaggio, 1990; Dequech, 2003) and the widely accepted scale development procedures proposed by Cope et al. (2016) and Hinkin (1998). On the other hand, to ensure culture relevance within the Chinese context, we adapted the items from research on Chinese traditional culture (Zhou et al., 2013; Zhang, 2024). The scale consists of four items measuring players' perception of the integration quality of ICH elements in the game, including diverse representations through environmental design, character development, visual aesthetics (costumes/skins), narrative and dialogue structures, musical style, and overall atmosphere, as well as alignment with core gameplay. Responses were measured on a 5-point Likert.

#### 2.3.2 Cultural identity attitude

The broad concept of cultural ethnic identity, equivalent to national identity, refers to an individual's self-identification as both a member of a specific ethnic group and a member of the nation in a multi-ethnic country. It is manifested through a sense of belonging and responsibility toward the nation, and also includes recognition, appreciation, and tolerance of other ethnic minorities. In psychological terms, identity is related to human mental activities and changes. Freud pointed out that the essence of identification is a shift in thinking patterns and behaviors. Hogg (2016) believes that the essence of cultural identity is the construct of attitudes (social identity theory); Ajzen (1991) believes that cultural identity is not only attitude but also reflected in cultural practice, symbolic consumption, and value practice, with attitude being the core precursor of behavior (planned behavior theory). Therefore, this paper studies cultural identity from two dimensions: attitudes toward cultural identity and cultural identity behaviors.

Cultural Identity Attitude refers to an individual's sense of belonging to and affection for traditional Chinese culture. Its measurement is adapted from a cultural identity scale developed by one Chinese scholar (Xiong, 2021), which assesses identification with traditional Chinese culture. The scale includes three items to assess changes in players' interest, appreciation, and pride in Chinese culture after engaging with the intangible cultural heritage-themed game, measured using a 5-point Likert scale.

#### 2.3.3 Cultural identity behavior

Cultural Identity Behavior refers to individuals' concrete actions that support the preservation and promotion of traditional Chinese culture. Its scale is adapted from the cultural identity scale developed by Xiong(2021), originally designed to assess behavioral engagement with traditional culture. The adapted scale consists of three items measuring increased willingness to share, learn about, and purchase elements of Chinese traditional culture after playing intangible cultural heritage-themed video games, all assessed on a 5-point Likert scale.

## 3 Data analyses result

### 3.1 Data statistical analyses

Table presents the participant characteristics and descriptive statistics for all measured variables. On average, participants had approximately 5 years of gaming experience (M = 4.82), played games about 3 times per week (M = 2.88), with a total weekly gaming duration of around 5 hours (M = 314.3 min). They regularly engaged with approximately 3 video games (M = 2.62), reported a longest continuous gaming session of about 6 h (M = 5.78), and had an average in-game spending of approximately 1,500 RMB (M = 1,663.74). In terms of demographics, 66.09% participants held an undergraduate or junior college degree, and 50.22% participants were current students. Regarding their Recognition of ICH, participants on average reported a moderate to slightly low level of understanding (M = 2.93).

In addition, descriptive statistics and correlation analyses were conducted for the three core variables: *ICH Embeddedness, Cultural Identity Attitude*, and C*ultural Identity Behavior*. Results showed that participants scored moderately high on all three variables: *ICH Embeddedness* (M = 3.81, SD = 0.90), *Cultural Identity Attitude* (M = 3.89, SD = 0.92), and C*ultural Identity Behavior* (M = 3.77, SD = 0.90). Correlation analysis indicated a significant positive association between *ICH Embeddedness* and *Cultural Identity Attitude* (*r* = 0.42, *p* < 0.01), as well as between *ICH Embeddedness* and *Cultural Identity Behavior* (*r* = 0.34, *p* < 0.01). Notably, a significant positive correlation was observed between Cultural Identity Attitude and Cultural Identity Behavior (*r* = 0.76, *p* < 0.01), indicating that stronger cultural attitudes are associated with more pronounced behavioral expressions of cultural identity.

### 3.2 The influence of the ICH embeddedness on cultural identity

Using the *ICH Embeddedness* in video games as the primary independent variable, we employed Ordinary Least Squares (OLS) regression to examine its impact on the ethnic cultural identity of young people (e.g., *Cultural Identity Attitude & Cultural Identity Behavior*). In particular, we also investigated a mediation model in which C*ultural Identity Attitude* mediates the ralationship between *ICH Embeddedness* and *Cultural Identity Behavior*. To account for potential confounding factors, we included a series of control variables, including participants' demographics (gender, age, income, education level, and occupation), gaming engagement characteristics (exposure to video games in terms of number, duration, and frequency, as well as the spending), and the participant's recognition of ICH. Regression results are presented in Table 1 as Analysis 1.

**Table 1:** The analysis results of embedding of ICH on cultural identity.

Analysis 1: step-by-step regression						
	Model (1)		Model (2)		Model (3)	
	Cultural identity behavior		Cultural identity attitude		Cultural identity behavior	
	*B*	*t*	*B*	*t*	*B*	*t*
ICH embeddedness	0.326^***^	7.662	0.418^***^	10.306	0.006	0.194
Cultural identity attitude					0.766^**^	23.54
Control variable	Controlled	Controlled	Controlled	Controlled	Controlled	Controlled
Constant	0.184	0.952	0.078	0.422	0.125	0.941
Adjusted *R*^2^	0.152		0.231		0.603	
*F* (*df*)	3.809		6.387		30.867	
Analysis 2: bootstrap method						
	Effect	Boot SE	Boot LLCI	Boot ULCI	Relative effect	
Direct effect	0.006	0.032	−0.057	0.07	1.90%
Indirect effect	0.32	0.042	0.236	0.403	98.10%

^**^p < 0.05. ^***^p < 0.01.

As shown in Model 1, *ICH Embeddedness* has a significant positive effect on *Cultural Identity Behavior* (*B* = 0.326, *t* = 7.662, *p* < 0.01), indicating that playing video games embedded with ICH elements can promote culture identity among young people. Using *Cultural Identity Attitude* as the independent variable, Model 2 shows that *ICH Embeddedness* also exerts a significant positive effect on *Cultural Identity Attitude* (*B* = 0.418, *t* = 10.306, *p* < 0.01). Model 3 includes *Cultural Identity Attitude* as an explanatory variable to check its mediation effects. The results show that *Cultural Identity Attitude* has a significant positive effect on C*ultural Identity Behavior* (*B* = 0.766, *t* = 23.540, *p* < 0.01), while the coefficient of *ICH Embeddedness* becomes non-significant (*B* = 0.006, *t* = 0.194, *p* > 0.05). These findings suggest that C*ultural Identity Attitude* serves as a mediation variable, between *ICH Embeddedness* and C*ultural Identity Behavior*.

To further validate the mediation effect, we employed the PROCESS macro developed by Hayes (2012) to test both direct and indirect effects using bootstrapping method. As shown in the Analysis 2 of Table 1, the 95% bootstrap confidence interval for the direct effect of *ICH Embeddedness* on *Cultural Identity Behavior* includes zero, indicating that the direct effect is not statistically significant. In contrast, the 95% bootstrap confidence interval for the indirect effect via *Cultural Identity Attitude* does not include zero, confirming a significant mediating effect. And the indirect effect (0.320) accounts for 98.10% of the total effect (0.326). These results provide robust evidence that *Cultural Identity Attitude* fully mediates the relationship between *ICH Embeddedness* and *Cultural Identity Behavior*.

### 3.3 Cluster analysis of young people

We employed the K-prototypes clustering algorithm to classify participants based on their demographic characteristics, gaming engagement variables. The analysis yielded three distinct clusters, accounting for 50.88%, 21.64%, and 27.49% of the sample, respectively. To examine the differences across the three clusters, we conducted ANOVA tests for the numerical variables and chi-square tests for categorical variables. As shown in Table 2, all variables exhibited statistically significant differences among the three groups (*p* < 0.05).

**Table 2:** Descriptive statistics for continuous and categorical participant characteristics and classification.

Numerical variables	Range	Mean ±SD	ANOVA test (Mean ± SD)			*P*
			Cluster 1 (*n* = 261)	Cluster 2 (*n* = 111)	Cluster 3 (*n* = 141)	
Game spending	500–5,000	1,663.74 ± 1,594.51	900.38 ± 945.45	2,216.22 ± 1,706.43	2,641.84 ± 1,748.80	0.000^**^
Weekly duration	15–1,200	314.30 ± 379.94	188.56 ± 212.27	595.41 ± 515.87	325.74 ± 378.01	0.000^**^
Weekly gaming frequency	1–7	2.88 ± 2.10	1.58 ± 1.04	5.31 ± 1.65	3.38 ± 1.98	0.000^**^
Number of frequently played games	1–5	2.62 ± 1.22	1.89 ± 0.70	4.00 ± 0.92	2.90 ± 1.12	0.000^**^
Total exposure time to games	1–8	4.82 ± 2.56	4.74 ± 2.40	6.14 ± 2.25	3.91 ± 2.57	0.000^**^
Maximum continuous gaming duration	1–24	5.78 ± 3.76	4.73 ± 3.24	7.72 ± 4.82	6.21 ± 2.94	0.000^**^
**Categorical variables**	**Options**	***N*** **(% of sample)**	Chi-square test (*n* (% of sample))			***P***
			**Cluster 1 (*****n*** = **261)**	**Cluster 2 (*****n*** = **111)**	**Cluster 3 (*****n*** = **141)**	
Age	18–21	224 (43.66)	157(60.15)	52(46.85)	15(10.64)	0.000^**^
	22–25	128 (24.95)	68(26.05)	30(27.03)	30(21.28)	
	26–28	91 (17.74)	17(6.51)	15(13.51)	59(41.84)	
	29–34	70 (13.65)	19 (7.28)	14 (12.61)	37 (26.24)	
Education level	Junior high school and below	33 (6.43)	10 (3.83)	4 (3.60)	19 (13.48)	0.000^**^
	High school	89 (17.35)	34 (13.03)	16 (14.41)	39 (27.66)	
	Bachelor degree	358 (69.79)	204 (78.16)	81 (72.97)	73 (51.77)	
	Master degree or above	33 (6.43)	13 (4.98)	10 (9.01)	10 (7.09)	

^**^p < 0.05.

Basing on the results in Table 2, the three clusters differ significantly in terms of age, gaming behavior, and three youth groups are outlined as follows:

Cluster 1 is characterized by low gaming engagement and younger age. Most individuals in this group are younger youth (18–25 years old), with education levels concentrated in undergraduate or junior college degrees. They report lower game spending, shorter playtime, and typically play 1–2 video games. Their exposure to video games is relatively recent, and their longest continuous gaming session tends to be shorter.

Cluster 2 is defined by high gaming engagement and younger age. This group also mainly consists of young individuals (18–25 years old) with similar educational backgrounds, but they exhibit higher spending on games, longer gaming time, regularly play 3–4 games, have longer exposure to video games, and report the longest continuous play duration among all clusters.

Cluster 3 represents individuals with moderate gaming engagement and older age. These participants are predominantly older youth (25–34 years old), similarly educated, and show the highest levels of in-game spending, relatively long gaming time, and regularly play 2–3 games. However, their overall exposure time to video games is shorter than that of Cluster 2, though they still report longer individual gaming sessions.

To further investigate whether different youth groups differ in their cultural identity behaviors, we conducted a one-way ANOVA on *Cultural Identity Behavior* across the three clusters. The results show that Cluster 1 had a mean score of 3.64 ± 0.84, Cluster 2 scored 3.89 ± 1.02, and Cluster 3 scored 3.91 ± 0.87, with the overall difference reaching statistical significance (*p* = 0.005). This suggests that *Cultural Identity Behavior* differs across the three clusters, with Cluster 2 and Cluster 3 scoring significantly higher than Cluster 1.

### 3.4 Heterogeneity among different youth groups

We introduced the youth groups—represented by the clusters identified in Section 3.3—as a moderating variable to examine the heterogeneity in the effect of *ICH Embeddedness* on *Cultural Identity Behaviors* across different groups. Basing on Model 1 in Table 1, we sequentially add the cluster and the interaction terms between clusters and *ICH Embeddedness* into the regression model. The results are reported in Table 3. As shown in Model 4, the cluster variable alone does not significantly affect *Cultural Identity Behavior*. However, Model 5 reveals that the interaction term between *ICH Embeddedness* and *Cluster 3* is significantly negative (*B* = −0.204, *t* = 2.025, *p* < 0.05). This indicates that, the positive effect of *ICH Embeddedness* on *Cultural Identity Behavior* for the youth in Cluster 3 is weaker than those in Cluster 1.

**Table 3:** Examination of the moderation of youth groups.

Variables	Model (4)		Model (5)	
	Cultural identity behavior		Cultural identity behavior	
	*B*	*t*	*B*	*t*
ICH embeddedness	0.325^**^	7.621	0.392^**^	6.109
Cluster 2	0.124	0.755	0.121	0.741
Cluster 3	0.177	1.319	0.198	1.468
Embedding Of ICH × Cluster 2			−0.016	−0.155
Embedding Of ICH × Cluster 3			−0.204^*^	−2.025
Control variables	Control	Control	Control	Control
Constant	0.106	0.512	0.116	0.563
Adjusted *R*^2^	0.155		0.163	
*F* (*df*)	3.572		3.492	

^*^p < 0.1. ^**^p < 0.05.

**Figure 1:**
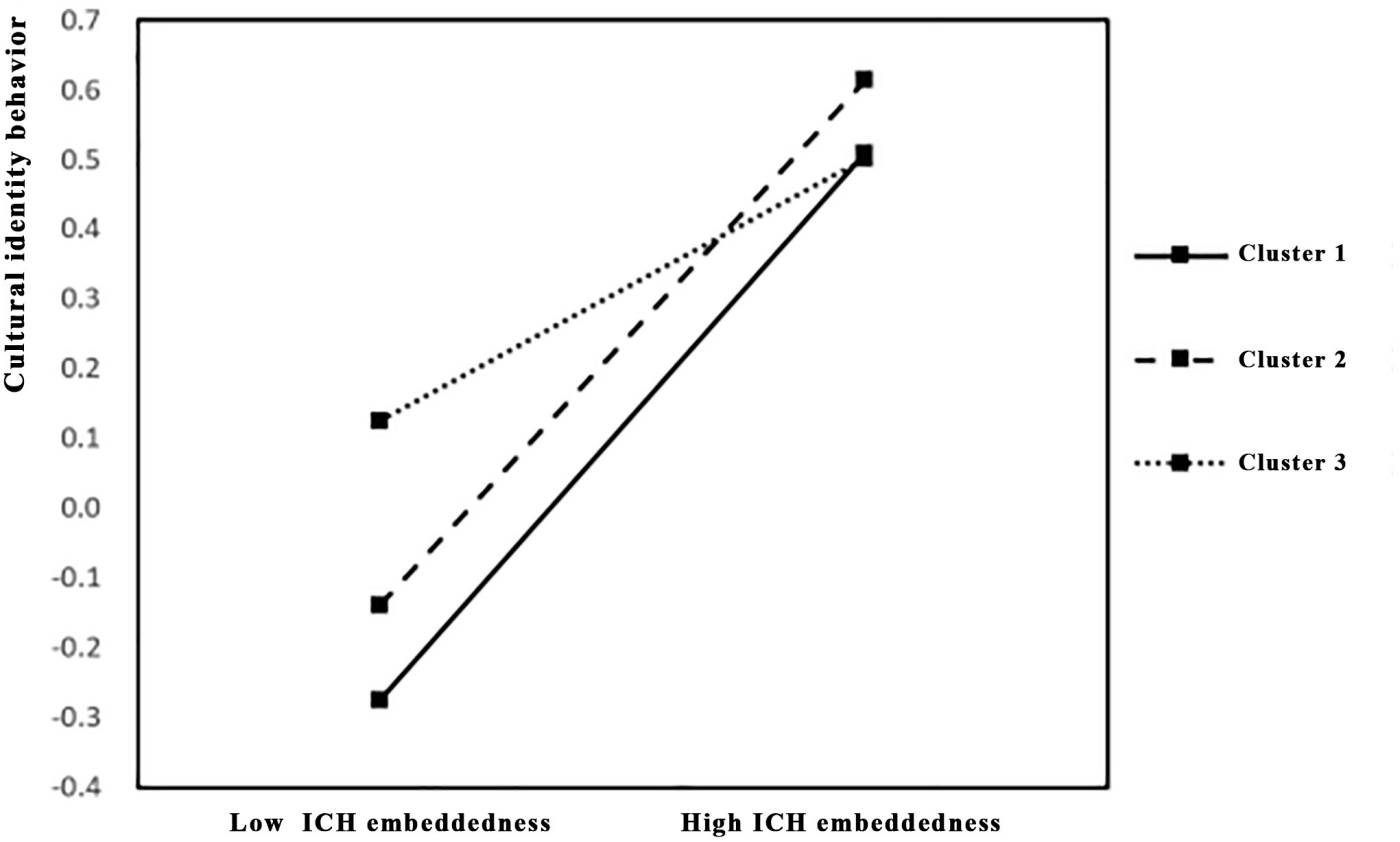
The moderating effect of different youth groups.

To further illustrate the moderating role of youth groups, we plotted the interaction effects across the three clusters, as shown in Figure 1. The slopes of the regression lines indicates that the influence of *ICH Embeddedness* on *Cultural Identity Behavior* is relatively strong for both Cluster 1 (*B*_1_ = 0.392) and Cluster 2 (*B*_2_ = 0.376), which primarily consist of younger players aged 18–25. In contrast, for Cluster 3 with mostly player aged 25–34, the effect is significantly weaker, with a slope of B_3_ = 0.1874 (*t* = 2.4273, *p* < 0.05), approximately half that of the other two clusters. This suggests that younger players are significantly more responsive to ICH video games than older youth.

Furthermore, Cluster 2, characterized by high gaming engagement, consistently exhibits the higher level of *Cultural Identity Behavior* than Cluster 1, which has low gaming engagement. As the *ICH Embeddedness* increases, the levels of *Cultural Identity Behavior* in Cluster 1 and Cluster 2 gradually catch up with or even surpass those of Cluster 3. It indicates that the younger youth (aged 18–25) can enhance their cultural identity through highly embedded ICH video games, potentially reaching a level comparable to that of older youth (aged 25–34).

## 4 Discussion

### 4.1 Findings

The three hypotheses proposed in this study all were verified, and their significance will be discussed below.

The main finding of this study demonstrates that integrating intangible cultural heritage (ICH) into video games is positively associated with the enhancement of cultural identity among young people. The deeper the ICH embeddedness, the stronger the game's cultural impact on players—affecting not only their cultural attitudes but also their cultural identity behaviors. This embeddedness is manifested through various culturally meaningful design elements, including traditional-inspired environments, character roles rooted in historical or mythological figures, ethnic costume aesthetics, heritage-based narratives and dialogue, and music incorporating traditional instruments or melodies. This may be attributed to the fact that video games create the information-rich environment that influences users' cognitive processes. According to Media Richness Theory, the richer a medium is, the more effectively it can facilitate the processing of uncertain and ambiguous information. Hyper-realistic scenes in games—enhanced by high-quality visual and interactive feedback—enable players to process cultural content more efficiently, thereby strengthening their sense of cultural identity (Tsadima et al., 2012; Zhang, 2024).

This study further provides evidence for the mediating role of cultural identity in the relationship between the ICH embeddedness and cultural identity behavior. From a dynamic perspective, embedded cultural values in games influence players' attitudes, which in turn shape their intentions and lead to cultural identity-related behaviors. These findings align with the Theory of Planned Behavior, which posits that attitude acts as a mediation between cultural exposure and behavioral change (Ajzen, 2011).

Moreover, this study reveals clear generational differences in the impact of ICH embeddedness on cultural identity, highlighting the moderating role of youth groups. The results show that younger players (aged 18–25) are significantly more responsive to video games deeply embedded with ICH. This may stem from their digital nativeness and greater receptiveness to immersive, game-based cultural learning (Korshunova, 2020). In contrast, older youth (aged 25–34) exhibit weaker responsiveness. This finding also align with Cognitive Development Theory, which holds that individuals in early youth are in a formative stage regarding cultural values, suggesting that early youth is a formative period for cultural identity construction (Deng et al., 2001), and that interactive, embodied engagement through games can serve as a powerful mechanism for cultural transmission (Mwangonde et al., 2021).

### 4.2 Implications

While existing research highlights the aging crisis in the inheritance of ICH (Wang et al., 2017), this study offers a practical solution: embedding ICH into video games to enhance cultural identity among young people. Rather than relying on simple content insertion, our findings highlights the importance of a “deep gamification” approach—integrating cultural elements meaningfully into game narratives, characters, aesthetics, and mechanics. To implement this strategy, cultural authorities are encouraged to establish long-term collaborations with game developers, promoting the integration of ICH into globally influential video games.

### 4.3 Limitations

The study also has limitations. First, this research relies on cross-sectional data, which limits the ability to examine the long-term impact and causal relationships of inheritance of intangible cultural heritage embeddedness on cultural identity. Longitudinal studies are needed to capture how sustained immersed in video games influences culture identity over time. Second, the sample is limited to the young people in China, restricting the generalizability of the findings across different cultural and social contexts. Future research should expand to include diverse populations from multiple countries to test the universality and boundary conditions of the observed effects. Additionally, future studies could explore other moderating factors, such as socioeconomic status or gaming preferences, to deepen the understanding of how different segments of youth engage with ICH content in video games.

## 5 Conclusion

This study finds that the embeddedness of ICH within video games significantly influences both cultural identity attitude and cultural identity behavior among young players. The influence of ICH embeddedness on cultural identity behavior is fully mediated by cultural identity attitude. Moreover, the strength of these effects is positively associated with the depth of ICH integration in the games. Notably, this influence is more pronounced among younger players (aged 18–25) and those exhibiting higher levels of gaming engagement, highlighting the moderating role of age and gaming involvement in the cultural transmission process. These findings contribute to a deep understanding of how video games serve as an effective medium for fostering cultural identity among young people.

## Data Availability

The raw data supporting the conclusions of this article will be made available by the authors, without undue reservation.
